# Supplementary material for: TAPBPR bridges UDP-glucose:glycoprotein glucosyltransferase 1 onto MHC class I to provide quality control in the antigen presentation pathway
Source: eLife. 2017 Apr 20;6:e23049. doi: 10.7554/eLife.23049 (PMC5441866; doi:10.7554/eLife.23049)
Supplement: Supplementary file 1. — To create the UDP-glucose:glycoprotein glucosyltransferase 1 binding site mutants, site-directed mutagenesis was performed on untagged TAPBPR in pCR-Blunt II-TOPO using Quik-Change site-directed mutagenesis (Stratagene) together with the primers specified in this table. The resultant TAPBPRUBS1 (I83K and E87K) and TAPBPRUBS2 (E87K, L90K, H91S, and D93R) variants were subsequently cloned into pHRSIN-C56W-UbEM and transduced into HeLaM cells. DOI: http://dx.doi.org/10.7554/eLife.23049.023 [file elife-23049-supp1.docx]

| Name | mutation | Primers used for site directed mutagenesis | Predicted TAPBPR domain |
| --- | --- | --- | --- |
| UBS1 | **I83K, E87K** | 5'-GCCTCAGTGGACCTGGTCCAGAAACCCCAGGCCAAGGCCTTGCTCCATGCTGACTGC-3'  5'-GCAGTCAGCATGGAGCAAGGCCTTGGCCTGGGGTTTCTGGACCAGGTCCACTGAGGC-3' | Unique N-terminal |
| UBS2 | **E87K, L90K**  **H91S, D93R** | 5'-GAAACCCCAGGCCAAGGCCTTGAAATCTGCTAGATGCAGTGGGAAGGAGGTGACC-3'  5'-GGTCACCTCCTTCCCACTGCATCTAGCAGATTTCAAGGCCTTGGCCTGGGGTTTC-3' |  |

Supplementary Table 1 – Primers used to generated TAPBPR^UBS1^ and TAPBPR^UBS2^ variant molecules
