# Supplementary material for: TAPBPR bridges UDP-glucose:glycoprotein glucosyltransferase 1 onto MHC class I to provide quality control in the antigen presentation pathway
Source: eLife. 2017 Apr 20;6:e23049. doi: 10.7554/eLife.23049 (PMC5441866; doi:10.7554/eLife.23049)
Supplement: Source code 1. — The maximum peak corresponding to B15 was aligned relative to that of the wild-type (WT) track because the separation of B15 and A68 is most distinctive. The separation point between B15 and A68 was identified in the WT track as the minimum between the two peaks. This distance between the B15 peak and the B15/A68 separation point as found in WT was calculated and applied to the alignment positions on the knockout and C94A tracks in order to separate B15 and A68 in these two tracks. Areas under the respective curves generated the densities of the corresponding MHC class I molecules. DOI: http://dx.doi.org/10.7554/eLife.23049.024 [file elife-23049-code1.docx]

TAPASIN.m

Calls im2curv.m

Script

A = imread('AAL2 test.gif');

image(A)

y = im2curv(A(16:268,:), 1, 1);

x = 1:length(y);

%%

minRange=[1 50;1 100;1 170];

n = size(minRange,1);

xm = zeros(1,n);

for k=1:n

[~, xm(k)]=min(y(minRange(k,1):minRange(k,2)));

end

xm = [x(1) xm x(end)];

z = y;

for k=2:n+2

L=xm(k-1):xm(k);

g = (y(xm(k))-y(xm(k-1)))/(L(end)-L(1));

z(L) = y(xm(k-1)) + (L-xm(k-1))*g;

end

dy = y - z;

[~,fpk] = max(dy(110:125)); fpk = fpk + 110 - 1;

[~,mx1]=min(dy(125:135));

mx1 = mx1 + 125 - 1;

subplot(312)

plot(x,dy,'-',[mx1 mx1],[0 dy(mx1)],'linewidth',1)

grid

S0=sum(dy(xm(2)+1:xm(3)));

S1=sum(dy(xm(3)+1:mx1));

S2=sum(dy(mx1+1:xm(4)));

disp([S0 S1 S2])

text(45,50,'TAPASIN')

text(110,50,'B15')

text(135,50,'A68')

text(45,20,strcat('(',num2str(S0,'%0.1f'),')'))

text(102,20,strcat('(',num2str(S1,'%0.1f'),')'))

text(130,20,strcat('(',num2str(S2,'%0.1f'),')'))

axis([1 length(x) 0 200])

title('Cy5 Densitometry (SNA)')

text(180,150,'WT')

ylabel('Intensity')

%%

y2 = im2curv(A(269:512,:), 1, 1);

x = 1:length(y2);

minRange2=[1 50;50 100;1 200;];

n = size(minRange2,1);

xm2 = zeros(1,n);

%%

for k=1:n

[~, s]=min(y2(minRange2(k,1):minRange2(k,2)));

xm2(k) = s + minRange2(k,1) - 1;

end

xm2 = [x(1) xm2 x(end)];

z2 = y2;

z2(xm2(1):xm(2))=y2(xm2(2));

for k=3:n+2

L=xm2(k-1):xm2(k);

g = (y2(L(end))-y2(L(1)))/(L(end)-L(1));

z2(L) = y2(L(1)) + (L-L(1))*g;

end

dy2 = y2 - z2;

[~,fpk2] = max(dy2(xm2(3):xm2(4))); fpk2 = fpk2 + xm2(3) - 1;

df12 = fpk - fpk2;

if df12<0

dy2 = [dy2(-df12+1:end) dy2(end)*ones(1,-df12)];

elseif df12>0

dy2 = [dy2(ones(1,df12)) dy2(1:end-df12)];

end

xm2(2:end-1) = max(1,min(xm2(end),xm2(2:end-1) + df12));

subplot(311)

plot(x,dy2,[mx1 mx1],[0 dy2(mx1)],'linewidth',1)

axis([1 length(x) 0 200])

grid

S0=sum(dy2(xm2(2)+1:xm2(3)));

S1=sum(dy2(xm2(3)+1:mx1));

S2=sum(dy2(mx1+1:xm2(4)));

disp([S0 S1 S2])

text(40,50,'TAPASIN')

text(106,50,'B15')

text(135,50,'A68')

text(40,20,strcat('(',num2str(S0,'%0.1f'),')'))

text(102,20,strcat('(',num2str(S1,'%0.1f'),')'))

text(130,20,strcat('(',num2str(S2,'%0.1f'),')'))

text(180,150,'KO')

ylabel('Intensity')

%%

y3 = im2curv(A(520:end-1,:), 1, 1);

x = 1:length(y3);

minRange3=[1 50;50 100;1 200];

n = size(minRange3,1);

xm3 = zeros(1,n);

%%

for k=1:n

[~, s]=min(y3(minRange3(k,1):minRange3(k,2)));

xm3(k) = s + minRange3(k,1) - 1;

end

xm3 = [x(1) xm3 x(end)];

z3 = y3;

z3(xm3(1):xm3(2))=y3(xm3(2));

for k=3:n+2

L=xm3(k-1):xm3(k);

g = (y3(L(end))-y3(L(1)))/(L(end)-L(1));

z3(L) = y3(L(1)) + (L-L(1))*g;

end

dy3 = y3 - z3;

[~,fpk3] = max(dy3(xm3(3):xm3(4))); fpk3 = fpk3 + xm3(3) - 1;

df13 = fpk - fpk3;

if df13<0

dy3 = [dy3(-df13+1:end) dy3(end)*ones(1,-df13)];

elseif df13>0

dy3 = [dy3(ones(1,df13)) dy3(1:end-df13)];

end

xm3(2:end-1) = max(1,min(xm3(end),xm3(2:end-1) + df13));

subplot(313)

plot(x,dy3,[mx1 mx1],[0,dy3(mx1)],'linewidth',1)

axis([1 length(x) 0 200])

grid

S0=sum(dy3(xm3(2)+1:xm3(3)));

S1=sum(dy3(xm3(3)+1:mx1));

S2=sum(dy3(mx1+1:xm3(4)));

disp([S0 S1 S2])

text(40,50,'TAPASIN')

text(106,50,'B15')

text(135,50,'A68')

text(40,20,strcat('(',num2str(S0,'%0.1f'),')'))

text(102,20,strcat('(',num2str(S1,'%0.1f'),')'))

text(130,20,strcat('(',num2str(S2,'%0.1f'),')'))

text(180,150,'C94A')

ylabel('Intensity')

xlabel('Gel Position')

im2curv.m

function y = im2curv( imdata, n, r0)

%IM2CURV Convert given image file to curves it contains

[r,c] = size(imdata);

dr = r / n;

y = zeros(n, c);

for k = 2:c-1

r1 = r0;

r2 = dr;

for f = 1:n

idx = find(~imdata(r1:r2,k),1);

if ~isempty(idx)

y(f,k) = dr-idx;

end

if ~y(f,k)

y(f,k)=y(f,k-1);

end

r1=r2+1;

r2=dr*f;

end

end

y=y(:,2:end-1);

end

TAPBPR.m

A = imread('AAL2 test.gif');

image(A)

%%

y = im2curv(A(16:340,:), 1, 1);

x = 1:length(y);

plot(x,y);

grid

%%

% [~,fpk] = max(y(100:120)); fpk = fpk + 100 - 1;

minRange=[30 100;300 400;400 460;460 500];

n = size(minRange,1);

xm = zeros(1,n);

for k=1:n

[~, s]=min(y(minRange(k,1):minRange(k,2)));

xm(k) = s + minRange(k,1) - 1;

end

xm = [x(1) xm x(end)];

z = y;

for k=2:n+2

L=xm(k-1):xm(k);

g = (y(xm(k))-y(xm(k-1)))/(L(end)-L(1));

z(L) = y(xm(k-1)) + (L-xm(k-1))*g;

end

dy = y - z;

plot(x,dy)

grid

%%

T = sum(dy(xm(2):xm(3)-1));

H = sum(dy(xm(5):xm(6)-1));

%%

y2 = im2curv(A(342:664,:), 1, 1);

plot(x,y2)

grid

%%

minRange2=[1 100;200 350;415 460;460 500];

n = size(minRange2,1);

xm2 = zeros(1,n);

for k=1:n

[~, s]=min(y2(minRange2(k,1):minRange2(k,2)));

xm2(k) = s + minRange2(k,1) - 1;

end

xm2 = [x(1) xm2 x(end)];

z2 = y2;

z2(xm2(1):xm(2))=y2(xm2(2));

for k=3:n+2

L=xm2(k-1):xm2(k);

g = (y2(L(end))-y2(L(1)))/(L(end)-L(1));

z2(L) = y2(L(1)) + (L-L(1))*g;

end

dy2 = y2 - z2;

plot(x,dy2)

grid

%%

T2 = sum(dy2(xm2(2):xm2(3)-1));

H2 = sum(dy2(xm2(5):xm2(6)-1));

%%

y3 = im2curv(A(666:end-1,:), 1, 1);

plot(x,y3)

grid

%%

minRange3=[1 100;300 400;420 470;470 500];

n = size(minRange3,1);

xm3 = zeros(1,n);

for k=1:n

[~, s]=min(y3(minRange3(k,1):minRange3(k,2)));

xm3(k) = s + minRange3(k,1) - 1;

end

xm3 = [x(1) xm3 x(end)];

z3 = y3;

z3(xm3(1):xm3(2))=y3(xm3(2));

for k=3:n+2

L=xm3(k-1):xm3(k);

g = (y3(L(end))-y3(L(1)))/(L(end)-L(1));

z3(L) = y3(L(1)) + (L-L(1))*g;

end

dy3 = y3 - z3;

plot(x,dy3)

grid

%%

T3 = sum(dy3(xm3(2):xm3(3)-1));

H3 = sum(dy3(xm3(5):xm3(6)-1));

%%

subplot(312)

plot(x,dy)

grid

Tstr = ['T = ',num2str(T)];

text(100,-4,Tstr)

Hstr = ['L = ',num2str(H)];

text(500,-4,Hstr)

text(300,250,'WT')

subplot(311)

plot(x,dy2)

grid

T2str = ['T = ',num2str(T2)];

text(100,-4,T2str)

H2str = ['L = ',num2str(H2)];

text(500,-4,H2str)

text(300,250,'KO')

subplot(313)

plot(x,dy3)

grid

T3str = ['T = ',num2str(T3)];

text(100,-4,T3str)

H3str = ['L = ',num2str(H3)];

text(500,-4,H3str)

text(300,250,'C94A')

set(gcf,'Position',[5 46 560 639])
